# Wide-spread brain alterations early after the onset of Crohn’s disease in children in remission—a pilot study

**DOI:** 10.3389/fnins.2024.1491770

**Published:** 2024-12-03

**Authors:** Pavel Filip, Lubomír Vojtíšek, Anna Marie Jičínská, Zdeněk Valenta, Ondřej Horák, Matěj Hrunka, Silvia Mangia, Shalom Michaeli, Petr Jabandžiev

**Affiliations:** ^1^Department of Neurology, First Faculty of Medicine and General University Hospital, Charles University, Prague, Czechia; ^2^Center for Magnetic Resonance Research (CMRR), University of Minnesota, Minneapolis, MN, United States; ^3^Department of Cybernetics, Czech Technical University in Prague, Prague, Czechia; ^4^Central European Institute of Technology (CEITEC) Masaryk University Neuroscience Centre, Brno, Czechia; ^5^Department of Paediatric Neurology, Faculty of Medicine, Masaryk University and University Hospital Brno, Brno, Czechia; ^6^Department of Statistical Modelling, Institute of Computer Science of the Czech Academy of Sciences, Prague, Czechia; ^7^Department of Paediatrics, University Hospital Brno, Faculty of Medicine, Masaryk University Brno, Brno, Czechia

**Keywords:** Crohn’s disease, diffusion tensor imaging, neuroinflammation, brain oedema, MRI relaxometry

## Abstract

**Background:**

The research on possible cerebral involvement in Crohn’s disease (CD) has been largely marginalized and failed to capitalize on recent developments in magnetic resonance imaging (MRI).

**Objective:**

This cross-sectional pilot study searches for eventual macrostructural and microstructural brain affection in CD in remission and early after the disease onset.

**Methods:**

14 paediatric CD patients and 14 healthy controls underwent structural, diffusion weighted imaging and quantitative relaxation metrics acquisition, both conventional free precession and adiabatic rotating frame transverse and longitudinal relaxation time constants as markers of myelination, iron content and cellular loss.

**Results:**

While no inter-group differences in cortical thickness and relaxation metrics were found, lower mean diffusivity and higher intracellular volume fraction were detected in CD patients over vast cortical regions essential for the regulation of the autonomous nervous system, sensorimotor processing, cognition and behavior, pointing to wide-spread cytotoxic oedema in the absence of demyelination, iron deposition or atrophy.

**Conclusion:**

Although still requiring further validation in longitudinal projects enrolling larger numbers of subjects, this study provides an indication of wide-spread cortical oedema in CD patients very early after the disease onset and sets possible directions for further research.

## Highlights

- Crohn’s disease is associated with brain changes already in the early phase of the disease.
- Advanced magnetic resonance imaging methods detected signs of oedema in brain areas important for cognition, behavior and control of autonomic bodily functions like heart rate, breathing and digestion.
- These brain changes indicate higher volume of brain cells in the cortex – a finding of possible association with autoinflammatory pathophysiology.

## Introduction

Crohn’s disease (CD), a transmural inflammatory disorder of gastrointestinal mucosa with the potential to affect the entire gastrointestinal tract, is a chronic debilitating condition with onset typically at a young age. Up to 25% of cases develop early during childhood and are associated with more severe course (Jabandziev et al., 2020a; Kelsen and Baldassano, 2008; Sỳkora et al., 2018; Van Limbergen et al., 2008). Approximately one third of CD patients develop extraintestinal manifestations (EIMs), predominantly affecting joints, skin, mouth, eyes and coagulation system (Rankin et al., 1979; Repiso et al., 2006), which either evolve parallel to the intestinal manifestations or precede their onset. Central nervous system (CNS) involvement has also been reported (Morís, 2014), with both neurological and psychiatric phenomena. Despite the clear need for timely diagnosis and management to prevent major component of morbidity (Wills et al., 2006) and the presence of neuropsychiatric complications suggested in up to one third of CD patients (Elsehety and Bertorini, 1997), there are not many studies investigating directly the affection of the CSN in CD.

The interaction between the brain and the gastrointestinal tract is a topic of growing interest, be it via psychological distress and related changes in the function of hypothalamic–pituitary adrenal (HPA) axis (Mackner et al., 2011; Mawdsley and Rampton, 2005), transmission of intestinal inflammatory signals to the brain (Bao et al., 2015) or even direct role of the abnormal central processing of the intestinal inflammatory response and dysregulation of the brain-gut axis in the pathogenesis of CD (Bonaz and Bernstein, 2013; Hollander, 2004). In general, visceral input is integrated in the central autonomic nervous system, relaying relevant information to endocrine, motor and behavioral processing nodes and multiple feedback loops controlling the sympathetic and parasympathetic outputs to peripheral organs, not excepting the immune system. Even a few imaging studies in CD patients have reported functional changes in several cortical and subcortical regions, including cingulate, insula and prefrontal cortex, associated with abdominal pain (Bao et al., 2016) or hypothesised as a general feature of CD (Bao et al., 2018). Interestingly, some of the functional alterations in the brain seemed to be sensitive to relevant therapeutic modalities utilised in CD as TNF-*α* (Gray et al., 2018). Moreover, further studies have detected structural changes in CD patients in remission, mainly increased volumes of multiple subcortical structures as hippocampus, basal ganglia and also thalamus (Bao et al., 2017; Nair et al., 2016). Nonetheless, such studies are burdened with substantial non-homogeneity in the disease duration and ages of included CD patients, an issue potentially associated with non-negligible bias, presence of further confounding factors as pain, anxiety and depression in CD patients, and restriction to one imaging modality only, precluding further deductions on the nature of the changes.

The presented pilot study focused on a highly homogeneous group of early onset CD adolescent patients in remission. Beyond the clear scientific interest in the effect of EIMs in developing brains particularly sensitive to any noxae of this nature, there was a clear unmet medical need – the confirmation of the presence of brain alterations associated with CD in paediatric population would be of profound implications for the clinical practice, calling for more complex management in cooperation with relevant specialists. Multimodal MRI protocol was implemented, combining cortical thickness and subcortical structure volume as macrostructural measures, along with several microstructural metrics. Diffusion tensor imaging (DTI) and neurite orientation dispersion and density imaging (NODDI) were implemented as microstructural parameters providing information about oedema, inflammation and tissue integrity (Kamiya et al., 2020). Furthermore, both conventional free precession longitudinal (T1) and transversal (T2) and adiabatic rotating-frame longitudinal (T1ρ) and transversal (T2ρ) relaxation time constants were measured. Conventional free precession relaxation metrics have a long-standing and well documented record of excellent sensitivity to myelin density and iron (Weiskopf et al., 2021). The ability of adiabatic rotating-frame relaxation metrics to detect slow motional regimens and different water dynamics ranges in kHz range, which are not readily visible to conventional protocols, should allow for more precise identification of eventual pathology. T1ρ has been previously associated with cellular loss (Michaeli et al., 2009) and T2ρ with iron loads (Mitsumori et al., 2009). Both parameters have had their utility confirmed under various pathologic conditions ranging from neurodegeneration (Filip et al., 2020a; Mangia et al., 2017) and autoimmune inflammatory conditions (Filip et al., 2021; Filip et al., 2020b) to ageing (Filip et al., 2023), largely exceeding the performance of more common MRI metrics.

Our primary hypothesis was rather broad due to the lack of previous data in the population in question. It postulated that early CD will be devoid of severe structural (atrophy) and major microstructural (large-scale iron content alterations, demyelination) cerebral changes due to relatively short time after the disease onset and only minor alterations will be detected such as cellularity or free water content differences, as expectable in autoimmune inflammatory conditions. Nonetheless, any structural/microstructural differences between CD patients and HC, even minor in extent, would indicate cerebral affection as an EIM developing already during the preclinical stage and/or higher impact of CD on CNS than initially expected.

## Methods

### Subjects

14 CD patients and 14 HC were enrolled into this pilot study. Relevant basic clinical data (clinical history, physical examination, laboratory and serological testing, imaging including MRI enterography and ileocolonoscopy and endoscopic examination with stepwise biopsy reviewed by a clinical pathologist) were obtained and evaluated. The following inclusion criteria were used for CD patients: the diagnosis of CD based on the ESPGHAN revised Porto criteria (Levine et al., 2014), ileocolonic location with both typical macroscopic and microscopic features of CD, evaluated by experienced gastroenterologist and pathologist, more than 2 years since disease onset, disease in clinical and laboratory remission for at least 6 months based on PCDAI score of <10 (Hyams et al., 2005) and faecal calprotectin values lower than 100 μg/g. Moreover, both CD patients and HC were deliberately chosen to be in the age group of 13–18 years. All CD patients were treated in accordance with national guidelines (Bronský et al., 2017). The exclusion criteria for both CD and HC groups were MRI contraindications in the study subject, claustrophobia, significant space occupying or vascular lesions in the MRI scans, comorbid neurological disorder, pregnancy. Every participant provided a written informed consent in accordance with the Declaration of Helsinki. The study protocol was approved by the ethics committee of the University Hospital Brno.

### Data acquisition—imaging protocol

The MRI acquisition was performed using a 3 Tesla Siemens Prisma system and the Siemens Prisma 64-channel head–neck coil at the Central European Institute of Technology in Brno, Czech Republic. The following metrics were acquired:

- T1-weighted (T1w) image and T1 map: magnetization-prepared 2 rapid gradient echoes (MP2RAGE) sequence in sagittal orientation with 1.0 mm isotropic resolution, echo time (TE) 2.98 ms, repetition time (TR) 5,000 ms, inversion times 700 ms (TI1) and 2,500 ms (TI2), flip angle 4° and 5°, generalized autocalibrating partial parallel acquisition (GRAPPA) acceleration factor of 3 (Marques et al., 2010).
- T2-weighted (T2w) image: SPACE sequence in sagittal orientation with 1.0 mm isotropic resolution, TE 412 ms, TR 3,200 ms, GRAPPA 2.
- Resting-state functional MRI (rs-fMRI): 2D multiband (MB) gradient-recalled echo (GRE) echo-planar imaging (EPI) sequence, 2.0 mm isotropic resolution, TR 820 ms, TE 35.2 ms, flip angle 45°, MB acceleration factor 4, consisting of 500 volumes. Two rs-fMRI runs were acquired with opposite phase encoding polarity [anterior–posterior (AP) followed by posterior–anterior (PA)]. Furthermore, a pair of spin echo images with AP and PA polarity utilizing a matching echo-spacing and resolution to the main rs-fMRI BOLD scans were acquired to allow for the correction of susceptibility-related distortions.
- Diffusion-weighted imaging (DWI): 1.5 mm isotropic resolution, TR 3,222 ms, TE 89.20 ms, MB acceleration factor 4, b-values of 1,500 and 3,000 s/mm2 in 93 directions, with 7 additional b0 images (Harms et al., 2018), acquired twice with opposite phase encoding polarity (AP and PA).
- T2 map: voxel size 1.0 × 1.0 × 3.5 mm, TR 7,020 ms, GRAPPA 2, with 17 echo times from 10 to 170 ms with ΔTE spacing of 10 ms.
- T1ρ and T2ρ: voxel size 1.6 × 1.6 × 3.5 mm, TR 2,000 ms, TE 2.82 ms, GRAPPA 3, preparation portion of the sequence adiabatic full passage hyperbolic secant pulses were used (Garwood and DelaBarre, 2001), adiabaticity factor R = 10, pulse duration = 6 ms, number of pulses = 0, 4, 8, 12, 16 phase cycled according to MLEV-4 (Levitt et al., 1969), bandwidth = 1.3 kHz, peak power ω_1_^max^/(2π) = 800 Hz.

All the sequences covered the entire brain including the cerebellum and brainstem.

### Image analysis

The image processing pipeline for structural T1w and T2w images was based on the human connectome project (HCP) minimal pre-processing pipeline (Glasser et al., 2013) with minor modifications. Specifically, the brain mask extraction process in the PreFreeSurfer step utilized the TI2 scan of the MP2RAGE sequence, the following steps were performed utilizing the TI1-TI2 combined T1w output of the MP2RAGE sequence (Marques et al., 2010). The FreeSurfer step was based on the CUDA (Compute Unified Device Architecture)-enabled version of FreeSurfer 6.0.^1^ HCP PostFreeSurfer step was implemented without any changes.

rs-fMRI acquisitions were utilized only for the multimodal cortical coregistration. The analysis consisted of the HCP minimal pre-processing pipeline and the HCP rsfMRI pipeline (Smith et al., 2013).

DWI processing also followed the HCP minimal pre-processing pipeline, including the optional gradient non-linearity correction. Afterwards, diffusion tensor model was fitted to generate fractional anisotropy (FA) and mean diffusivity (MD) maps. DWI scans with the *b*-value of 3,000 mm/s^2^ were not utilized for diffusion tensor fitting to avoid the deviation from the mono-exponential model of intravoxel incoherent motion. Furthermore, Bingham-NODDI analysis was performed to extract voxel-wise intracellular volume fraction (fICVF) and total orientation dispersion index (ODI).

T1ρ, T2ρ and T2 maps were generated as follows: after 3D rigid-body motion correction of all the acquired scans to the first scan of each of these sequences (trilinear interpolation, mutual information as cost function followed by an optimization pass with sinc interpolation as implemented in the FSL 6.0 MCFLIRT), relaxation time constants were calculated utilizing 2-parameter non-linear fitting (custom routines in MATLAB R2019; MathWorks, Inc., Natick, MA). In the next step, each of the maps was co-registered to the native “HCP space”.

For all the microstructural parameters considered in the analysis (MD, FA, fICVF, ODI, T1, T2, T1ρ, and T2ρ map), subcortical grey matter (GM) structures, including cerebellum were then warped to the MNI space using the matrices calculated in the HCP minimal preprocessing pipeline. Cortical voxels were mapped to the individual cortical surfaces utilizing partial-volume-weighted ribbon-constrained volume-to-surface mapping algorithm and resampled to the standard greyordinate space. These cortical reconstructions and cortical thickness maps (with regressed-out linear effects of curvature) were then warped utilizing the “MSMAll” multimodal areal feature-based surface registration algorithm (Glasser et al., 2016; Robinson et al., 2014) and combined with subcortical GM images to create CIFTI files. Cortical thickness maps were combined with volumes of subcortical structures normalised by the individual estimated intracranial volume. In the last step, the data were parcellated utilizing combined HCP-derived cortical parcellation consisting of 180 parcels per hemisphere (Glasser et al., 2016) and resting-state network based sub-segmentation of Freesurfer-derived subcortical grey matter structures using Cole-Anticevic Brain Network Atlas (Ji et al., 2019), with a lower threshold of 50 voxels for each subcortical segment size, yielding 68 subcortical regions of interest (ROIs).

All the data processing outputs were visually evaluated by a trained operator (P.F.). Root-mean-squared voxel displacement both in DWI and rs-fMRI data was checked. Due to the young age of the participants, less stringent criteria for motion (3-voxel threshold during the whole acquisition of DWI and rs-fMRI separately) were implemented.

### Statistical analyses

Demographic data were summarised descriptively; two-sample, two-tailed T-test and Fisher’s exact test was used for the comparison of age and sex, respectively, between CD and HC. Two separate general linear models (GLMs) were run, both with MRI data in CIFTI format (cortical thickness + subcortical volumes, T1, T2, T1ρ and T2ρ map, MD, FA, fICVF, ODI) as the dependent variable and the following independent variables:

1. Study group for the comparison between HC and CD patients;
2. The disease duration before the treatment start as the predictor for the evaluation of clinical metrics (based only on the 14 CD patients).

Permutation-based non-parametric analysis as implemented in the Permutation Analysis of Linear Models package (Winkler et al., 2014) was used, with 10,000 permutations. Statistical significance level of 0.05 was adopted with false discovery rate (FDR) correction (Benjamini and Hochberg, 1995) within each modality, one-sided tests are reported to distinguish the direction of the difference between CD and HC. Cortical clustering threshold of 3 was implemented to exclude singleton cortical parcels.

## Results

There were no statistically significant differences in the age (*p* = 0.390) and sex (*p* = 0.704) distributions of CD (average [SD] – 15.7 [1.3] years; 7 females) and HC (15.3 [1.3] years; 5 females) (see Table 1 for basic clinical data on CD).

**Table 1:** Basic clinical information on patients with Crohn’s disease.

#	Sex	Age		Paris classification	Therapy		
		At the diagnosis	At MRI acquisition		First-line	Second-line	Maintenance (at MRI acquisition)
1	F	12y 2mo	15y 2mo	A1bL3B1G1	EEN + TP	CS	TP
2	M	15y 2mo	17y 6mo	A1bL3B1G0	EEN + TP	none	TP
3	M	12y 2mo	14y 2mo	A1bL3B3pG0	IFX + TP	none	IFX + TP
4	M	14y 8mo	17y 6mo	A1bL3B1G0	EEN + TP	CS	TP
5	M	14y 6mo	17y 5mo	A1bL3B1G0	EEN + TP	CS	ADA
6	F	14y 3mo	16y 7mo	A1bL3B1G1	EEN + TP	CS	TP
7	F	13y 2mo	15y 6mo	A1bL3B1G0	EEN + TP	CS	IFX + TP
8	M	12y 4mo	15y 4mo	A1bL3L4aB1G0	EEN + TP	CS	TP
9	F	10y9mo	17y1mo	A1bL3B1G0	EEN + TP	none	TP
10	F	9y6mo	15y6mo	A1aL3B1pG1	EEN + TP	none	TP
11	M	12y10mo	17y9mo	A1bL3B1G0	GCS + TP	CS	TP
12	F	15y9mo	17y11mo	A1bL3B1G0	CDED+TP	none	TP
13	M	10y10mo	13y9mo	A1bL3B1G1	EEN + TP	CDED	IFX + TP
14	F	13y1m	15y9m	A1bL3B1G0	GCS + TP	CS	IFX + TP

F – female; M – male; y – years; mo – months; EEN – exclusive enteral nutrition; TP – thiopurine; GCS – glucocorticosteroids; IFX – infliximab; ADA – adalimumab; CDED – Crohn’s Disease Exclusion Diet.

**Figure 1:**
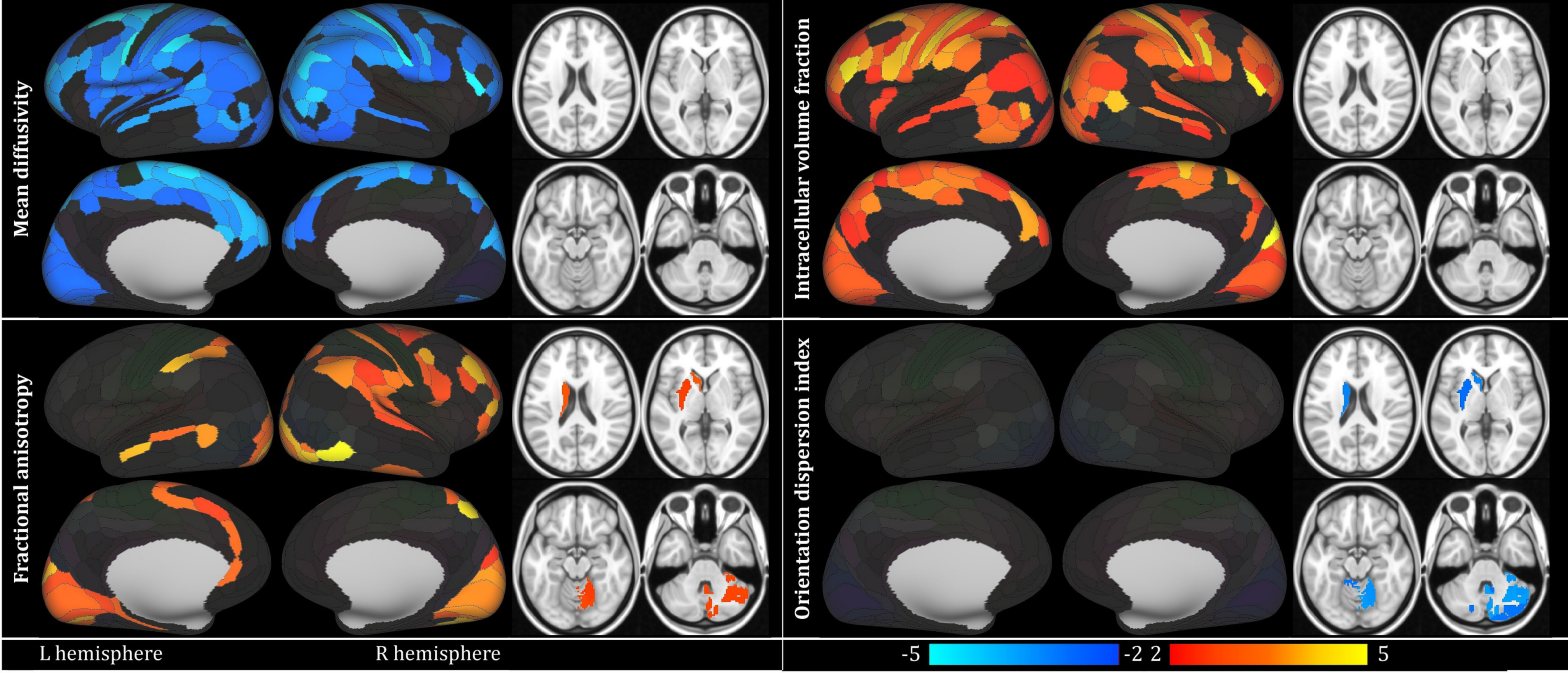
Main results of the comparison between patients with Crohn’s disease (CD) and healthy controls (HC) presented only for metrics detecting statistically significant results: mean diffusivity, fractional anisotropy, intracellular volume fraction and orientation dispersion index. Alpha of 0.05, within-modality false discovery rate corrected was implemented. Red-yellow scale marks CD > HC contrast, blue scale labels the reverse contrast; values correspond to the T statistic. Subcortical structures shown in 4 slices z = 20, 2, −16, −34 (MNI coordinate system). Laterality convention where the right side of the figure corresponds to the right side of the brain is used. No statistically significant findings in subcortical structures for mean diffusivity and intracellular volume fraction, and in cortical structures for orientation dispersion index. See Table 2 for full anatomical and statistical information on significant regions. L – left; R – right. For the full list of abbreviations and information on the parcellation, see Glasser et al. (2016).

No statistically significant inter-group differences were detected in the cortical thickness and the relaxation metrics. The analysis of DWI data revealed wide-spread cortical inter-group differences, with lower MD and higher fICVF in the CD group over bilateral frontal, parietal and also occipital cortices, but not in the subcortical grey matter (see Figure 1 and Table 2, Supplementary Table S1 for detailed information, including group means and standard deviations, effect size, T statistic and *p* values). Furthermore, higher FA in the CD group was detected in both primary and secondary visual cortices, but also right-side premotor and prefrontal cortex, bilateral parietal lobe both in the superior and inferior parietal cortex, and in the lateral temporal cortex. Furthermore, FA was higher in the CD group in the left-side striatum and the right cerebellum. And lastly, ODI failed to detect any inter-group differences in the cortex, but yielded similar subcortical pattern of grey matter affection – left-side striatum and predominantly right cerebellum.

**Table 2:** Main results of the comparison between patients with Crohn’s disease (CD) and healthy controls (HC) presented only for metrics detecting statistically significant results: mean diffusivity (values multiplied by the factor of 1,000 for better legibility), fractional anisotropy, intracellular volume fraction and orientation dispersion index.

	Side and anatomical area		#	Crohn’s disease	Healthy controls	Effect size	T stat	*p* value (FDR)
Mean diffusivity CD < HC	L	Dorsolateral Prefrontal, Inferior Parietal, Paracentral Lobular and Mid Cingulate, Anterior Cingulate, Premotor, Superior Parietal and IPS, Inferior Frontal, Insular, Visual cortices, Posterior Operculum, Auditory Association, Orbital and Polar Frontal, Somatosensory and Motor, Temporal–Parietal-Occipital Junction, Lateral Temporal, Posterior Cingulate	97	0.610 [0.007]	0.619 [0.008]	1.135	−5.995	0.004
	R	Inferior Parietal, Dorsolateral Prefrontal, Superior Parietal and IPS, Visual cortices, Premotor, Paracentral Lobular and Mid Cingulate, Somatosensory and Motor, Temporal–Parietal-Occipital Junction, Inferior Frontal, Orbital and Polar Frontal, Auditory Association, Posterior Operculum, Anterior Cingulate, Lateral Temporal	70	0.614 [0.008]	0.623 [0.008]	1.130	−5.813	0.004
Fractional anisotropy CD > HC	R	Inferior Parietal, Superior Parietal and IPS, Auditory Association, Early Auditory, Paracentral Lobular and Mid Cingulate, Posterior Operculum, Somatosensory and Motor, Temporal–Parietal-Occipital Junction	15	0.146 [0.004]	0.137 [0.005]	1.768	4.632	0.007
	R	Dorsolateral Prefrontal, Inferior Frontal, Premotor, Orbital and Polar Frontal	12	0.145 [0.004]	0.139 [0.004]	1.680	4.183	0.007
	R	Visual cortices, Lateral Temporal	14	0.142 [0.004]	0.133 [0.006]	1.624	5.356	0.007
	R	Premotor, Posterior Operculum	4	0.153 [0.005]	0.146 [0.005]	1.603	3.974	0.007
	L	Lateral Temporal, Auditory Association	3	0.145 [0.006]	0.135 [0.006]	1.560	4.046	0.007
	L	Superior Parietal and IPS, Inferior Parietal	4	0.142 [0.005]	0.134 [0.006]	1.390	4.113	0.007
	L	Anterior Cingulate, Paracentral Lobular and Mid Cingulate	5	0.156 [0.004]	0.149 [0.006]	1.362	3.157	0.013
	L	Visual cortices, Medial Temporal, Posterior Cingulate	10	0.146 [0.005]	0.138 [0.007]	1.308	4.106	0.007
	R	Cerebellum	4	0.200 [0.011]	0.191 [0.011]	0.843	2.817	0.023
	L	Caudate	1	0.174 [0.033]	0.154 [0.009]	0.806	3.075	0.023
	L	Putamen	1	0.176 [0.016]	0.166 [0.013]	0.708	2.698	0.029
fICVF CD > HC	R	Superior Parietal and IPS, Inferior Parietal, Dorsolateral Prefrontal, Visual cortices, Paracentral Lobular and Mid Cingulate, Premotor, Inferior Frontal, Somatosensory and Motor, Posterior Operculum, Temporal–Parietal-Occipital Junction, Orbital and Polar Frontal, Posterior Cingulate	63	0.399 [0.009]	0.389 [0.008]	1.085	6.453	0.004
	L	Dorsolateral Prefrontal, Superior Parietal and IPS, Inferior Parietal, Paracentral Lobular and Mid Cingulate, Premotor, Visual cortex, Somatosensory and Motor, Orbital and Polar Frontal, Posterior Cingulate, Anterior Cingulate, Auditory Association, Temporal–Parietal-Occipital Junction, Lateral Temporal, Posterior Operculum	82	0.399 [0.009]	0.389 [0.009]	1.055	5.788	0.004
	R	Auditory Association	3	0.388 [0.009]	0.380 [0.010]	0.806	2.942	0.010
	R	Auditory Association, Lateral Temporal	3	0.383 [0.008]	0.377 [0.008]	0.804	3.565	0.004
	L	Insular	3	0.384 [0.010]	0.377 [0.010]	0.682	2.442	0.028
ODI CD < HC	R	Cerebellum	5	0.438 [0.020]	0.462 [0.025]	1.063	−3.608	0.029
	L	Caudate	1	0.514 [0.067]	0.555 [0.019]	0.836	−3.189	0.043
	L	Cerebellum	1	0.448 [0.022]	0.466 [0.024]	0.763	−2.910	0.046
	L	Putamen	1	0.545 [0.037]	0.568 [0.022]	0.742	−2.830	0.040

Data reported as clusters, with cortical anatomical localisation based on 22 main cortical segments and parcellation as defined by Glasser et al. (2016), number of parcels contained in each cluster (column #), average and standard deviation separately for CD and HC group, effect size (Cohen’s d), T statistic of permutation-based T test and *p* value. Alpha of 0.05, within-modality False Discovery Rate correction was implemented. Entries sorted in descending order based on the effect size. See also Figure 1 and Supplementary Table S1 for more information. L – left; R – right; T stat – T statistic; FDR – False Discovery Rate; fICVF – intracellular volume fraction; ODI – orientation dispersion index.

**Figure 2:**
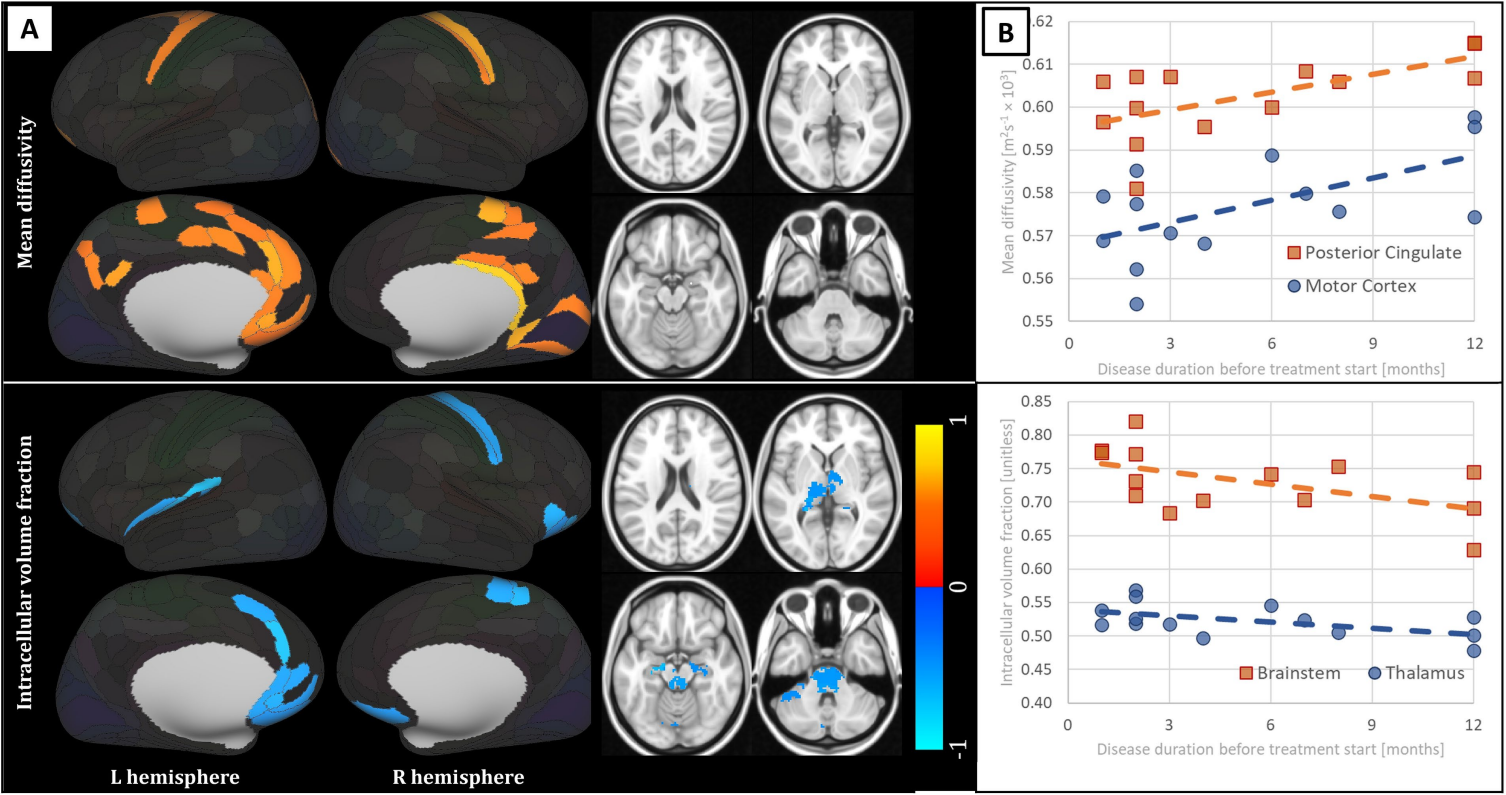
Results of the analysis of correlation of disease duration before the treatment initiation in patients with Crohn’s disease and MRI parameters, presented only for metrics detecting statistically significant results: mean diffusivity and intracellular volume fraction. **(A)** Anatomical distribution of the findings. Red-yellow scale marks positive correlation, blue scale the reverse contrast; values correspond to the R statistic. Alpha of 0.05, false discovery rate corrected was implemented. Subcortical structures shown in 4 slices z = 20, 2, −16, −34 (MNI coordinate system). Laterality convention where the right side of the figure corresponds to the right side of the brain is used. No statistically significant findings in subcortical structures for mean diffusivity. **(B)** Scatterplots of relevant MRI metrics (x axis – months of disease duration, y axis – respective MRI metric) over relevant regions of interest with linear trendlines overlaid. See Table 3 for full anatomical and statistical information on significant regions. L – left; R – right.

In the analysis correlating MRI parameters with the disease duration before the treatment start, there was a positive correlation of MD in the posterior cingulate and motor cortex and negative correlation of fICVF in the thalamus, brainstem and frontal pole area (see Figure 2; Table 3).

**Table 3:** Results of the analysis of correlation of disease duration before the treatment initiation in patients with Crohn’s disease and MRI parameters, presented only for metrics detecting statistically significant results: mean diffusivity (values multiplied by the factor of 1,000 for better legibility) and intracellular volume fraction.

	Side and anatomical area		#	R stat	*p* value (FDR)
MD positive correl.	R	Posterior Cingulate, Early Visual	6	0.728	0.010
	L	Dorsal Stream Visual	3	0.665	0.010
	R	Paracentral Lobular and Mid Cingulate, Somatosensory and Motor	4	0.632	0.016
	L	Anterior Cingulate, Orbital and Polar Frontal, Dorsolateral Prefrontal, Paracentral Lobular and Mid Cingulate	13	0.624	0.010
	L	Posterior Cingulate, Superior Parietal and IPS	3	0.594	0.011
	L	Premotor, Somatosensory and Motor	3	0.543	0.024
fICVF negative correlation	L	Early Auditory, Insular	4	−0.703	0.016
	L	Hippocampus (Visual2 Somatomotor)	2	−0.682	0.016
	L	Anterior Cingulate, Orbital and Polar Frontal	9	−0.640	0.016
	R	Somatosensory and Motor, Paracentral Lobular and Mid Cingulate	3	−0.595	0.016
	L	Thalamus (Visual Visual2 Somatomotor Cingulo-Opercular)	4	−0.578	0.020
	R	Orbital and Polar Frontal, Insular	5	−0.561	0.016
	C	Brainstem (Visual Visual2 Somatomotor Posterior Multimodal)	4	−0.542	0.025
	L	Diencephalon ventral (Visual)	1	−0.516	0.032
	R	Thalamus (Cingulo-Opercular Frontoparietal)	2	−0.484	0.044
	R	Hippocampus (Somatomotor)	1	−0.460	0.049
	L	Cerebellum (Dorsal Attention)	1	−0.455	0.049

Data reported as clusters, with cortical anatomical localisation based on 22 main cortical segments and parcellation as defined by Glasser et al. (2016), number of parcels contained in each cluster (column #), R statistic and *p* value. Alpha of 0.05, within-modality False Discovery Rate corrected was implemented. Entries sorted in descending order based on the R statistic. See also Figure 2. L – left; R – right; C – central; R stat – R statistic; FDR – False Discovery Rate; MD – mean diffusivity; fICVF – intracellular volume fraction.

## Discussion

We are presenting the outcomes of a multimodal brain imaging study in a highly homogeneous group of early onset CD patients in remission. Particularly the combination of the short time after the onset of disease symptoms with metrics providing information on the macrostructure and microstructure of the brain allows for constructive inferences. While no structural alterations or differences in conventional and adiabatic metrics as proxies of myelination, cellular loss and iron density were detected, extensive cortical areas exhibited inter-group differences in MD and fICVF – parameters previously associated with cytotoxic oedema and neuroinflammation (Kamiya et al., 2020; Sevick et al., 1992; Loubinoux et al., 1997). Although of major clinical importance given the highly sensitive period of brain development during childhood and adolescence (Fuhrmann et al., 2015), the issue of large-scale microstructural impairment of the central nervous system associated with this chronic autoimmune disease has eluded more substantial attention of both researchers and clinicians. Ergo, considering the relative rarity of CD in this group, this study presents important novel information calling for further research and caution in clinical settings.

The absence of structural cerebral alterations in early CD is of no surprise given the short disease course and absence of substantial neurological problems in this study group. However, rather intriguing is the lack of findings in all the relaxation metrics. Both conventional and adiabatic longitudinal and transversal relaxation are generally heralded as highly sensitive measures of tissue microstructure, albeit with limited specificity to precise underlying pathophysiology. Adiabatic T1ρ and T2ρ have been repeatedly shown to outperform other more conventional metrics in the detection of the supposed pathological or physiological changes, be it in neurodegeneration (Mangia et al., 2017; Mangia et al., 2014), demyelination (Filip et al., 2021; Filip et al., 2020b) or ageing (Filip et al., 2023). Nonetheless, the absence of any statistically significant inter-group differences in these metrics must be viewed in the context of substantial widespread findings in DWI-derived metrics. MD has long been known as a highly sensitive and viably specific marker of tissue oedema, where a highly similar metric – apparent diffusion coefficient – has penetrated into the common clinical practice in various medical fields. Lower diffusivity values have been previously associated with cytotoxic oedema (Sevick et al., 1992; Loubinoux et al., 1997), i.e., a condition with increased intracellular volume. This finding corresponds well to the higher intracellular volume fraction (fICVF) in CD with exceedingly similar cortical spatial distribution (see Figure 1). And although the utility of NODDI and the viability of the underlying model has been rightly questioned under pathological conditions and in grey matter (Guerrero et al., 2019), there is a large body of research supporting the relevance of this parameter in various relevant disease models (Kamiya et al., 2020). On the other hand, FA in cortical GM is known to be associated with unmyelinated large-calibre dendrites rather than myelin density or cellularity in general (Reveley et al., 2022; Preziosa et al., 2019). Interestingly, the subcortical findings in FA virtually mirror the inter-group differences in ODI, even though their laterality would likely be a result of insufficient statistical power than the true underlying nature of the pathology itself. And lastly, both MD and fICVF exhibited significant correlations with disease duration before the treatment start. Values of both metrics in CD patients with shorter duration of the disease, were more distant from the levels detected in HC, i.e., lower in MD and higher in fICVF. Whether this partially counter-intuitive finding is to be explained by much more aggressive disease course in these patients leading to earlier consultation of relevant physicians and therapy start, remains to be evaluated in further studies. However, the distribution of datapoints where the disease duration before diagnosis was shorter than 3 months in nearly half of the subjects calls for caution against over-zealous interpretation of these results.

Also the distribution of the detected alterations is of much interest—the affection of bilateral insular cortex, dorsolateral prefrontal cortex but also subcortical GM structures, including upper brainstem and hypothalamus (see Figures 1, 2), is well in accord with the hypothesised maladaptation of the HPA axis (Bonaz and Rivest, 1998) and the position of stress as a major culprit involved in local inflammatory responses in the gastrointestinal tract (Taché and Bonaz, 2007; Tache and Perdue, 2004), also directly in CD (Straub et al., 2002). Prefrontal and insular cortices have been reported to regulate peripheral immune cells via autonomic and neuroendocrine pathways (Tracey, 2002) and parasympathetic tone (Bonaz and Bernstein, 2013). Hence, the imbalance between the adaptive capacities of the body, also at the level of the CNS, may sway the equilibrium between the HPA axis and the autonomous nervous system (Chrousos, 2009), as also seen in idiopathic bowel diseases in general (Straub et al., 2002). Also the detected affection of the anterior cingulate, dorsolateral and dorsomedial cortex, nodes linked to major depressive disorder (Bora et al., 2012), seems in line with the literature on CD, as increased prevalence of depression and anxiety have been repeatedly reported in this population, even in remission (Farrokhyar et al., 2006; Kim et al., 2013).

Furthermore, therapeutical modalities utilised in the studied group may be of importance as well. Specifically the administration of corticosteroid second-line treatment in three quarters of the includes CD patients, even though temporary, could be related to the alterations detected in microstructural metrics, given the non-negligible effect of corticoids on several parts of limbic system even in short-term applications (Joëls et al., 2013). However, none of the study subjects used corticosteroids at the time of the acquisition, decreasing the probability of such influences. The maintenance treatment based on thiopurine is generally not considered to elicit substantial central nervous system alterations, although in long-term therapy, several possible effects have been hypothesised (Sutton et al., 2019). All in all, the size of the included cohort, absence of longitudinal data and substantial diversity of therapeutic approaches do not allow for further inferences on the potential central nervous system effects of therapy in CD. Further studies will definitely find the interplay of developing brain and the presented pathophysiological processes of interest, even though necessitating cautious approach in the design to account for non-negligible inter-individual variability in paediatric population.

Nonetheless, several limitations of the study must be recognized. Firstly, the relatively high sensitivity of implemented MRI metrics to CNS tissue alterations is offset by low specificity to the actual nature and aetiology of the detected changes. While hypercoagulability and/or vasculitis-related thromboembolism, immunologic abnormities, malabsorption, pharmacological side effects and infections have been suggested as potential aggressors in CD (Morís, 2014; Gobbele et al., 2000; Zois et al., 2010) leading to cerebrovascular events, depression, anxiety and fatigue (Stovicek et al., 2014; Thomann et al., 2016), currently available MRI protocols do not allow us to provide a full and complex overview of the pathophysiological nature of the perceived affection. The cross-sectional character of the study also precludes more precise implications due to both disease and subject related variability. Secondly, the low number of enrolled subjects required non-negligible concessions in the statistical approach, including the lack of further regressors in GLMs accounting for eventual effects of age and sex, and over-arching higher-level correction for multiple comparisons over all the MRI metrics implemented. Although it is partly forgivable by the relative scarcity of the investigated condition (Jabandziev et al., 2020b) when complying with the strict inclusion criteria to ensure as high homogeneity as possible, it will definitely affect the strength of our conclusions. In the light of problems with reproducibility of neuroscientific studies (Button et al., 2013) and even calls for the decrease of generally accepted thresholds for statistical significance (Benjamin et al., 2018), we certainly caution against overconfidence in the numerical results of the study and ignoring as of now unmodelled uncertainties about the disease itself. Nonetheless, this research project was conceived as a pilot study to show possible directions for further research and to substantiate subsequent well-designed, multi-centric, longitudinal studies in an area largely neglected in the scientific literature, hence vindicating the lower statistical power (Lakens et al., 2018).

## Conclusion

Although still requiring further validation in longitudinal studies enrolling larger numbers of subjects, this study indicates large-scale cortical cytotoxic oedema and alterations of dendritic structure in CD patients very early after the disease onset, pointing to their development already during the preclinical stage and/or higher propensity of CD for the central nervous system than previously anticipated, affecting nodes essential for the regulation of the autonomous nervous system, sensorimotor processing, cognition and behavior. This association between CD and brain should definitely be kept in mind even in clinical practice, especially in the highly sensitive period of brain development during childhood and adolescence.

## Data Availability

The raw data supporting the conclusions of this article will be made available by the authors, without undue reservation.

## Footnotes

^1^ http://surfer.nmr.mgh.harvard.edu
